# Supplementary figures and images for: Leucine rich amelogenin peptide prevents ovariectomy-induced bone loss in mice
Source: PLoS One. 2021 Nov 15;16(11):e0259966. doi: 10.1371/journal.pone.0259966 (PMC8592471; doi:10.1371/journal.pone.0259966)

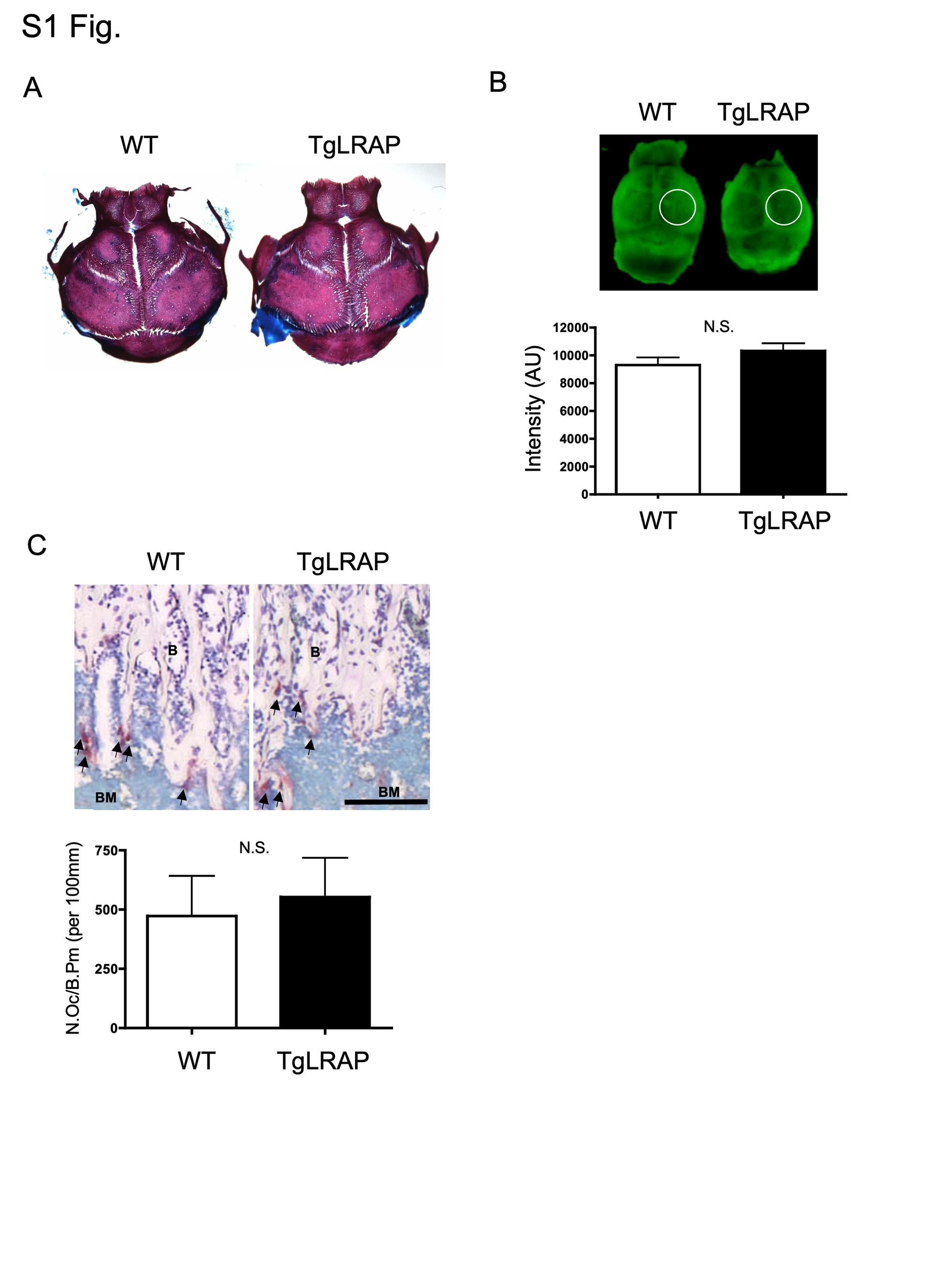

Supplement: S1 Fig — A, Alizarin red /alcian blue double staining of 5-day-old calvaria. No change in bone development was apparent. B, Evaluation for bone formation. The calcein intake was compared between WT and TgLRAP. A white circle with 4 mm diameter was used for region of interest (ROI). Green color shows areas of high osteoblast activity and dark color shows those of low osteoblast activity. No significant difference was observed. AU; arbitrary unit. Each column in graphs represents the mean ± SD. n = 5–6 for each group. N.S.; not significant. C, TRAP staining of tibial metaphysis. The number of osteoclasts in secondary spongiosa was counted and compared between WT and TgLRAP samples. The osteoclast number was not significantly different between the groups. B; bone, BM; bone marrow, Arrows; osteoclasts. Bar = 100 μm. Each column in graphs represents the mean ± SD. n = 5–6 for each group. N.S.; not significant. (TIF) [file pone.0259966.s001.tif]

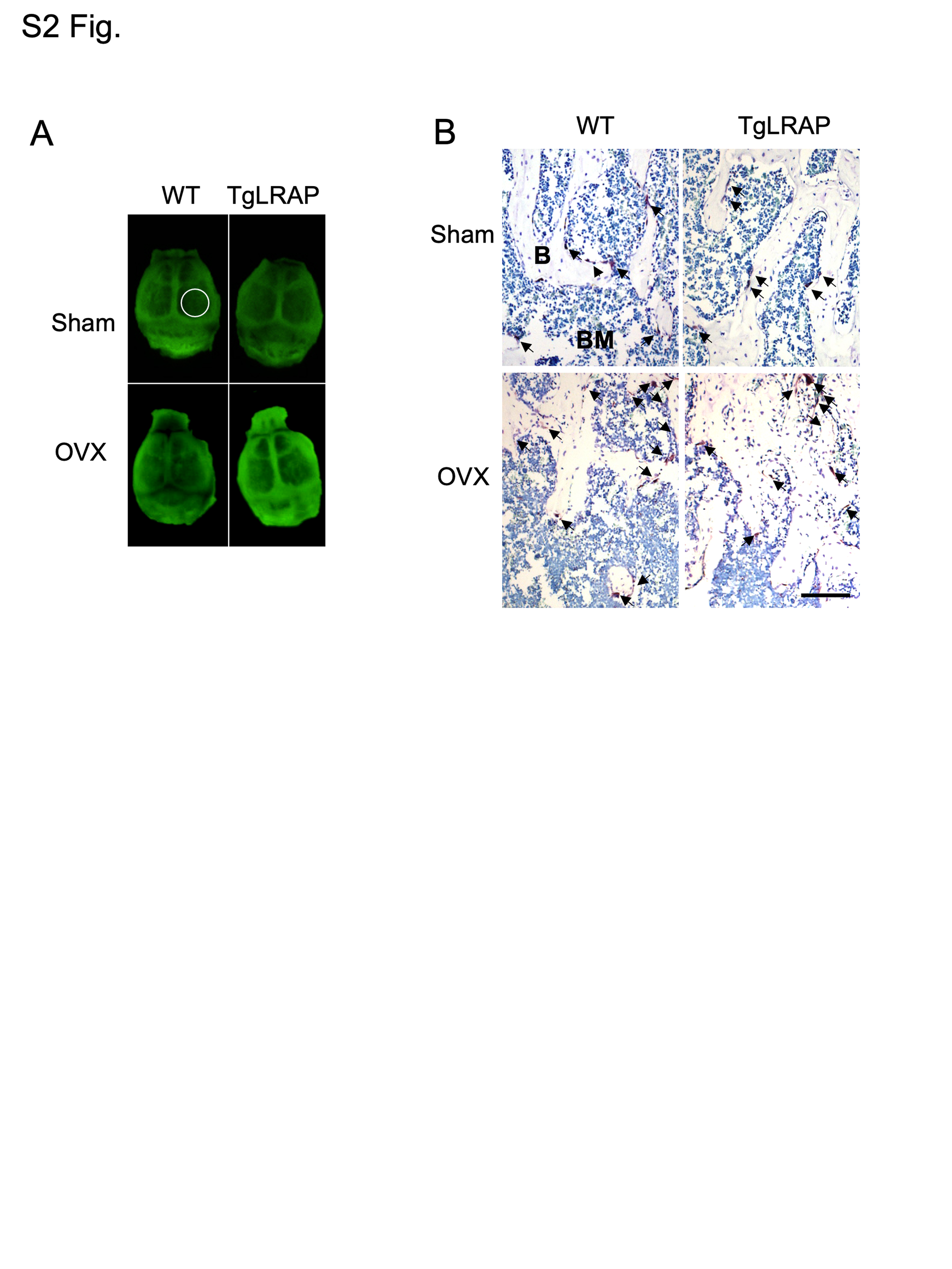

Supplement: S2 Fig — A, Representative examples for quantitation of calcein incorporation into the calvaria. B, Representative examples of TRAP staining on Sham /OVX WT or TgLRAP mice femur. TRAP positive cells are indicated by arrows. B, bone; BM, bone marrow. Bar = 50 μm. (TIF) [file pone.0259966.s002.tif]

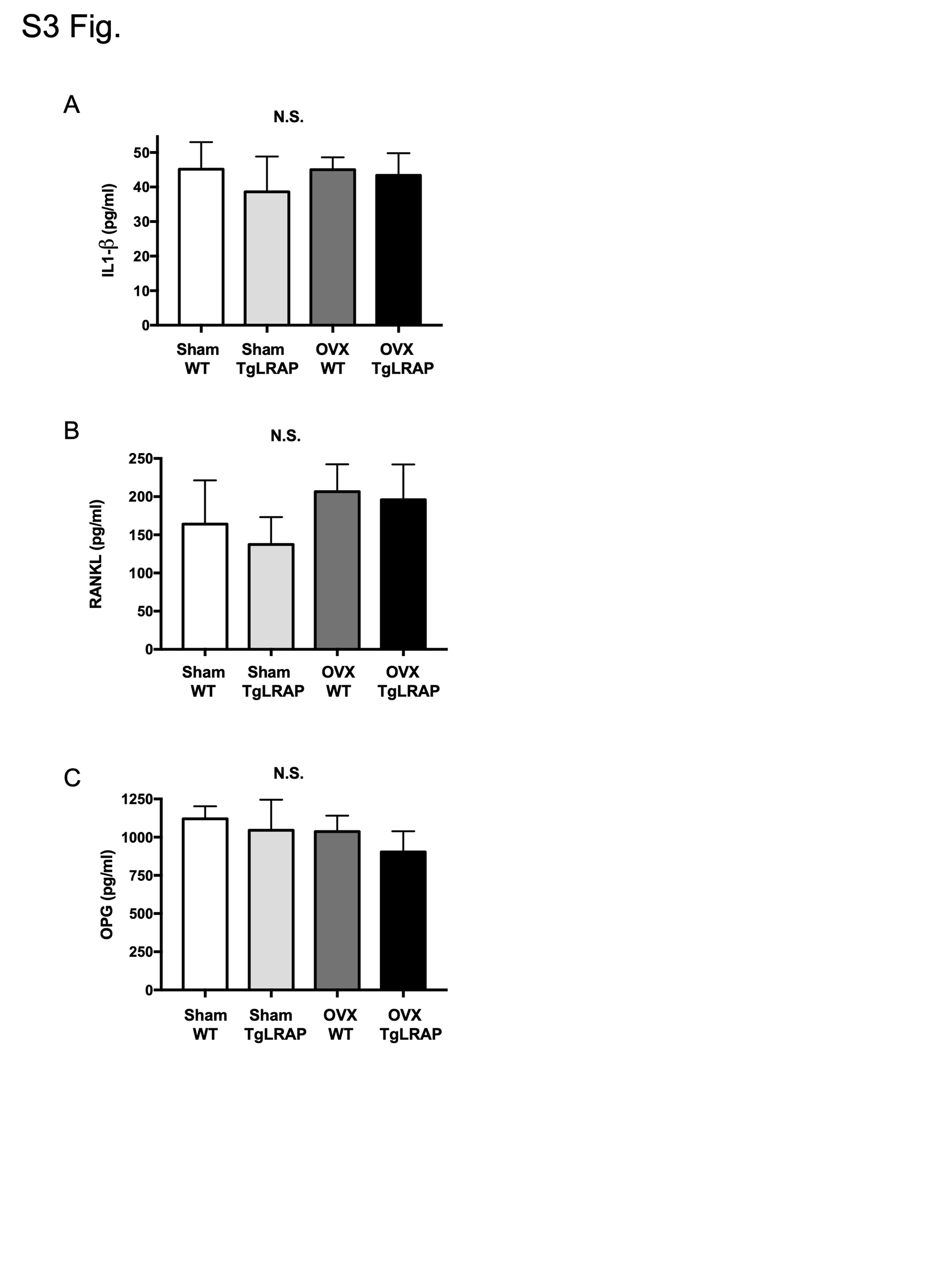

Supplement: S3 Fig — A, IL-1β. B, RANKL. C, OPG. Each column in graphs represents the mean ± SD. n = 4–6 for each group. N.S.; not significant. (TIF) [file pone.0259966.s003.tif]

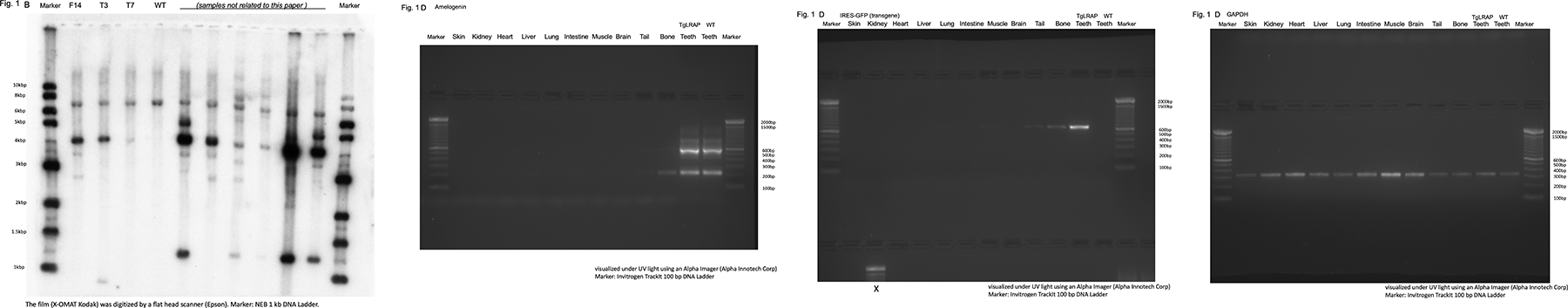

Supplement: S4 Fig — The first image in S4 depicts a raw image for Fig 1B and next three images depict raw images for Fig 1D 3 subpanels. (TIF) [file pone.0259966.s004.tif]
