# Supplementary material for: Leucine rich amelogenin peptide prevents ovariectomy-induced bone loss in mice
Source: PLoS One. 2021 Nov 15;16(11):e0259966. doi: 10.1371/journal.pone.0259966 (PMC8592471; doi:10.1371/journal.pone.0259966)
Supplement: S1 Table — (PDF) [file pone.0259966.s005.pdf]

S1 Table

Sequences for target gene primers

PRIMER	*SEQUENCES*	*GenBank accession*
Amelx (F)	5-AATGGGGACCTGGATTTTGTTG-3	NM_009666
Amelx (R)	5-TCCCGCTTGGTCTTGTCTGTCGCT-3	
Genotyping (F)	5-GGATTTTGTTTGCCTGCCTCC-3	NM_009666
Genotyping (R)	5- CACTTCTTCCCGCTTGGTCTTG-3	
IRES-EGFP (F)	5-CCACCATATTGCCGTCTTTTG-3	-
IRES-EGFP (R)	5-CCACAACTATCCAACTCACAAC-3	
Gapdh (F)	5-CCATCACCATCTTCCAGGAG-3	NM_001001303
Gapdh (R)	5-GCATGTgLRAPGACTGTGGTCATGAG-3	
Oc (F)	5-CTGACCTCACAGATCCCAAGC-3	NM_031368
Oc (R)	5-TGGTCTGATAGCTCGTCACAAG-3	
Opg (F)	5-ACCCAGAAACTGGTCATCAGC-3	NM_008764
Opg(R)	5-CTGCAATACACACACTCATCACT-3	
Alp (F)	5-CCAACTCTTTTGTGCCAGAGA-3	NM_007431
Alp (R)	5-GGCTACATTGGTGTTGAGCTTTT-3	
Opn (F)	5-AGCAAGAAACTCTTCCAAGCAA-3	NM_009263
Opn (R)	5-GTGAGATTCGTCAGATTCATCCG-3	
Rankl (F)	5-CAGCATCGCTCTGTTCCTGTA-3	NM_011613
Rankl (R)	5-CTGCGTTTTCATGGAGTCTCA-3	
Runx2 (F)	5-CCAACCGAGTCATTTAAGGCT-3	NM_009820
Runx2 (R)	5-GCTCACGTCGCTCATCTTG-3	
Mmp13 (F)	5-CTTCTTCTTGTTGAGCTGGACTC-3	NM_008607
Mmp13 (R)	5-CTGTGGAGGTCACTGTAGACT-3	
Hprt (F)	5-TCAGTCAACGGGGGACATAAA-3	NM_013556
Hprt (R)	5-GGGGCTGTACTGCTTAACCAG-3	
Bsp (F)	5-CAGGGAGGCAGTGACTCTTC-3	NM_008318
Bsp (R)	5-AGTGTGGAAAGTGTGGCGTT-3	
